# Characterization and Molecular Interpretation of the Photosynthetic Traits of *Lonicera confusa* in Karst Environment

**DOI:** 10.1371/journal.pone.0100703

**Published:** 2014-06-24

**Authors:** Geng Wu, Haibo Jia, Yongwei Huang, Lu Gan, Chunhua Fu, Libin Zhang, Longjiang Yu, Maoteng Li

**Affiliations:** 1 School of Life Science and Technology, Huazhong University of Science and Technology, Wuhan, Hubei, China; 2 State Key Laboratory of Biogeology and Environmental Geology, China University of Geosciences, Wuhan, Hubei, China; Key Laboratory of Horticultural Plant Biology (MOE), China

## Abstract

*Lonicera confusa* was a medical plant which could adapt to the Ca-rich environment in the karst area of China. The photosynthesis, relative chlorophyll content,differentially expressed genes (DEGs) and differentially expressed proteins (DEPs) of *L. confusa* that cultivated in calcareous and sandstone soils were investigated. The results showed that the relative chlorophyll content and net photosynthesis rate of *L. confusa* in calcareous soil are much higher than that planted in sandstone soil, the higher content of calcium might play a role in keeping the chloroplast from harm and showed higher photosynthesis rate. The transpiration and stomata conductance were decreased in calcareous soil, which might result from the closure of stomata. The GeneFishing and proteomic results showed that the expression of DEGs and DEPs were critical for photosynthesis and stomata closure, such as RuBisCO, photosynthetic electron transfer c and malate dehydrogenase varied in the leaves of *L. confusa* that cultivated in different soils. These DEGs or DEPs were further found to be directly or indirectly regulated by calcium sensor proteins. This study enriched our knowledge of the molecular mechanism of high net photosynthesis rate and lower transpiration of *L. confusa* that cultivated in the calcareous soil in some degree.

## Introduction

The highly ordered and complex process of plant leaf development is influenced by a variety of factors and constrained by several abiotic stresses. The research on the mechanisms of the adaptation to abiotic stress environment in plants is becoming a hotspot in recent years [1]–[3]. Carbonate rock is the bedrock of karst ecosystems, which occupies about 1/3 of mainland of China [4],the high calcium content and drought are the main characteristics of karst soils [5]. Accordingly, the plants in karst areas have obtained the ability to adapt to the high level of calcium and drought through long-term evolution [6]–[8]. Research on the mechanisms of plant adaptation to karst environments is necessary for it can provide new ways on the fragile karst ecosystem rehabilitation. *L. confusa* was one of Chinese medicinal plant with a typical ecological value and which was widely cultivated in calcium-rich karst areas of China [9]. Previous studies had showed that the cytosolic free Ca^2+^ could restrict to 10^−7^ M or less in *L. confusa* [10], because of it could excrete the excess of Ca^2+^ via stomata or stored in glands and trichomes under higher Ca^2+^ supplied in karst areas [8].

As the key process for neogenesis of biological material, photosynthesis plays a central role in plant performance under abiotic stresses, and the net photosynthesis rate (Pn) and transpiration rate (E) in most fruit crops could be reduced with a rapid closure of stomata, with the reduction of stomata conductance (Gs) as well [11]–[13]. As one of essential and major plant nutrients, Ca^2+^ is required to maintain cell wall structure and membrane function [14]. Soil Ca^2+^ depletion could affect some important physiological processes, such as carbohydrate storage, photosynthesis, chlorophyll content and antioxidant enzyme activity [15], [16]. It was revealed that the decline of photosynthesis caused by the simulated acid rain treatment could be recovered with high concentration of Ca^2+^ treatment in *Lonicera formosana* [17]. Ca^2+^ are directly involved in several aspects of photosynthesis through modulating phosphatase enzymes activity and regulating chloroplast activity of NAD^+^ kinase [18]. Tan *et al* (2011) revealed the photosynthesis is improved by exogenous calcium treatment in heat-stressed tobacco [19]. Research on photosynthesis of plants that adapt to karst areas was performed in recent years, Huang et al (2006) have studied the photosynthesis, transpiration and water use efficiency of pioneer specie *Cornus controversa*, *Zenia insigni* and *Lonicera maackii* in karst area, the results showed that these 3 karst species can process a physiecological drought and thermal adaptation and develop a strategy to escape environmental stress [20], but the molecular mechanisms of photosynthesis in *L. confusa* has not been mentioned [20], [21].

In this paper, the variation in net photosynthetic rate (Pn), relative chlorophyll content (Chl), stomata conductance (Gs) and transpiration rate (E) were evaluated in *L. confusa* that cultivated on calcareous and sandstone soils, respectively. The differentially expressed genes (DEGs) and differentially expressed proteins (DEPs) were identified in the meanwhile by GeneFishing PCR and two-dimensional gel electrophoresis (2-DE) in order to explain the molecular mechanism of *L. confusa* that cultivated in different calcium cultivation conditions.

## Results

### Comparative analysis of Chl content, Pn, E and CI in leaves of *L. confusa* that cultivated in calcareous and sandstone soils

**Figure 1:**
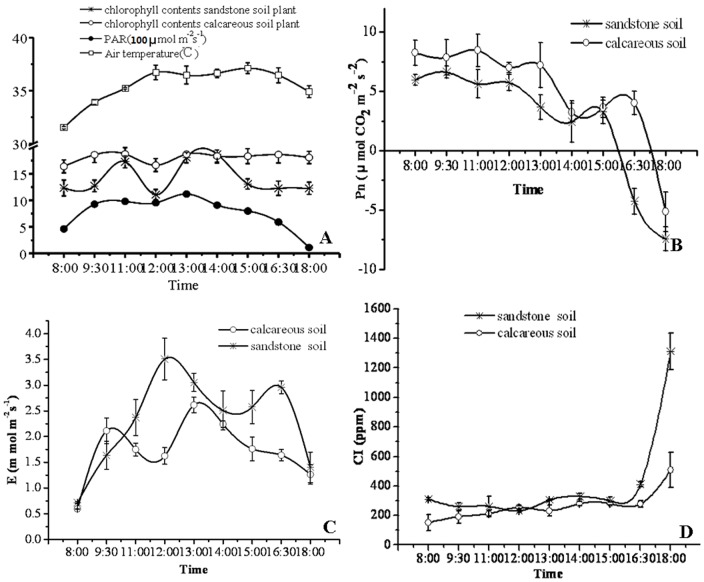
Diurnal variation of environment factors, chlorophyll content, Pn, E, CI. A. Diurnal variation of chlorophyll content, PAR, air temperature; B, C and D represent the Diurnal variation of CI, Pn and E, respectively.

The diurnal variation of environment factors was exhibited in Fig. 1. The average photosynthetic active radiation (PAR) and air temperature were 762.2 µ mol m^−2^ s^−1^ and 35.4°C, respectively. The PAR value was increased continually from 461.2 µmol m^−2^ s^−1^ to 1119.0 µmol m^−2^ s^−1^ from 08:00 to 13:00 and decreased to 111.7 µmol m^−2^ s^−1^ at 18:00. The air temperature was up to highest point at 13:00 (37.12°C) and down at 15:00 (Fig. 1A).

**Figure 2:**
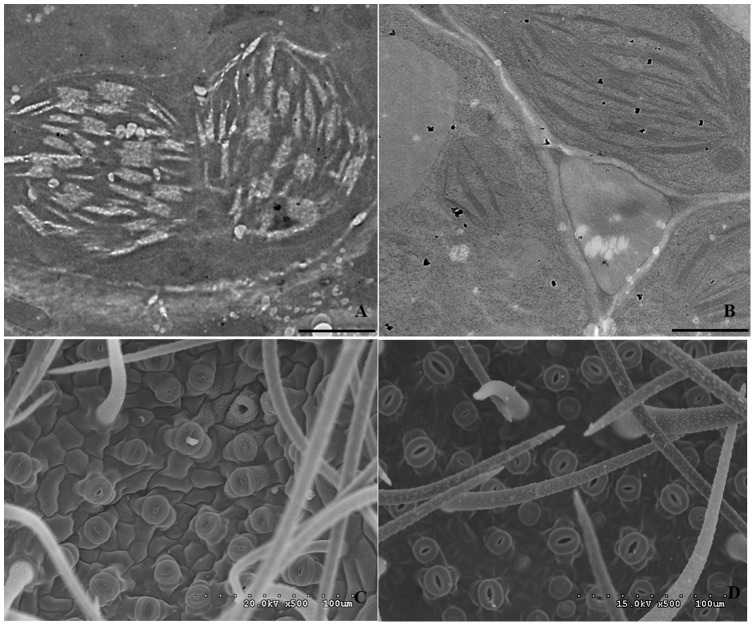
Observation of stomata status by using SEM and Ca^2+^ positioning analysis chelated by EGTA by using TEM. A and B represent the Ca^2+^ positioning analysis in the chloroplast of leaves that cultivated in calcareous soil and sandstone soil that chelated by EGTA, Bar in A and B represent 1000 nm; C and D represent the SEM observation of stomata status of leaves that cultivated in calcareous soil and sandstone soil.

Chlorophyll (Chl) is the molecule in photosynthesis that absorbs sunlight and uses its energy to synthesize carbohydrates from CO_2_ and H_2_O. Present results revealed that the relative content of Chl in *L. confusa* planted in calcareous soil was higher than that of planted in sandstone soil (Fig. 1A). Further research indicated that the relative Chl contents in two cultivated soils were both decreased to 11.1 and 13.8 at 12:00 noon, and after that time relative content of Chl was increased to 18.1 and 18.0, respectively. It also revealed that the relative Chl content in calcareous soil was relatively stable. Conversely, the relative content of Chl in sandstone soil varied a lot in diurnal, it indicated that the relatively higher calcium supply in calcareous soil is of importance for maintaining the stability of the chlorophyll. Further TEM observation revealed that more small bubble appeared in chloroplast grana of *L. confusa* leaves planted in calcareous soil when the TEM sections were treated with calcium chelator EGTA (Fig. 2A). Conversely, very few bubble appeared in the chloroplast of *L. confusa* that cultivated in sandstone soil (Fig. 2B). These results directly suggested the content of Ca^2+^ in chloroplast of leaves that cultivated in calcareous soil was much higher than that of *L. confusa* leaves planted in sandstone soil, which might keep the chloroplast unharmed under strong solar radiation. The Pn of *L. confusa* in calcareous soil was significantly higher than that cultured in sandstone soil (Fig. 1B), which indicated that *L. confusa* cultivated in calcareous soil might synthesize more carbohydrates daily. From 08:00 to 12:00, the Pn in two cultivated condition was maintained relatively stable and the tendency as a whole decreased after 12:00. The highest Pn of *L. confusa* in calcareous soil was found at 11:00. However, the highest Pn was observed at 9:30 in sandstone soil, and both represent at a lowest value at 14:00. The phenomena mentioned above suggested the relatively higher calcium supply in calcareous soil could improve the Pn of *L. confusa*, which was consistent with the higher relative Chl content in the leaves of *L. confusa* that planted in calcareous soil.

The E of *L. confusa* represented a typical double peak curve under the two culture conditions (Fig. 1C). When cultured in calcareous soil, the E of *L. confusa* arose rapidly from 8:00 AM and the double peak appeared at 9:30 AM and 1:00 PM with the value of 2.1 mmol m^−2^ s^−1^ and 2.6 mmol m^−2^ s^−1^, respectively. However, the double peak appeared at 12:00 and 14:30 with the value of 3.5 mmol m^−2^ s^−1^ and 2.9 mmol m^−2^ s^−1^ respectively, when cultivated in sandstone soil. Further research revealed that the average value of E in *L. confusa* that cultivated in calcareous soil was 1.73 mmol m^−2^s^−1^, which was lower than that in sandstone soil (2.17 mmol m^−2^s^−1^). As we know, the E value were regulated by the degree of stomatal opening state, the stomatal closure would reduce the E value to decrease the water loss. The stomata conductance (Gs) analysis showed that the average Gs was 93.08 mmol m^−2^ s^−1^ and 117.45 mmol m^−2^s^−1^ under calcareous soil and sandstone soil, respectively. SEM analysis showed that most of stomata in the leaves of *L. confusa* were closed in the leaves planted in calcareous soil, otherwise, half of the stomata were in normal status in the leaves planted in sandstone soil (Fig. 2C, 2D), which was in accordance with E value of *L. confusa* planted in sandstone soil was higher than that of *L. confusa* planted in calcareous soil. The intercellular CO_2_ concentration (CI) revealed that the average CI of *L. confusa* planted in calcareous soil was lower than that planted in sandstone soil (Fig. 1D), which was also consistent with the E value mentioned above.

The correlation between E, Gs, Pn were analyzed and showed in Table S3. On the conditions of sandstone, the Pn of *L. confusa* were significantly positive correlated with PAR (p<0.05) and significantly negative correlated with CI (p<0.01), other environment factors has little effect on Pn, no obvious correlation was found between the E, Gs and environment factors (Table S3). Otherwise, the E and Gs of calcareous soil planted *L. confusa* were significantly correlated with PAR, air temperature and leaf temperature, the Pn was also shown positive correlation with PAR (Table S3). All those results indicated that the *L. confusa* in relative higher level of calcium could enhance its sensitivity to environmental factors.

### The identification of DEGs and DEPs through GeneFishing PCR and 2-DE analysis

**Figure 3:**
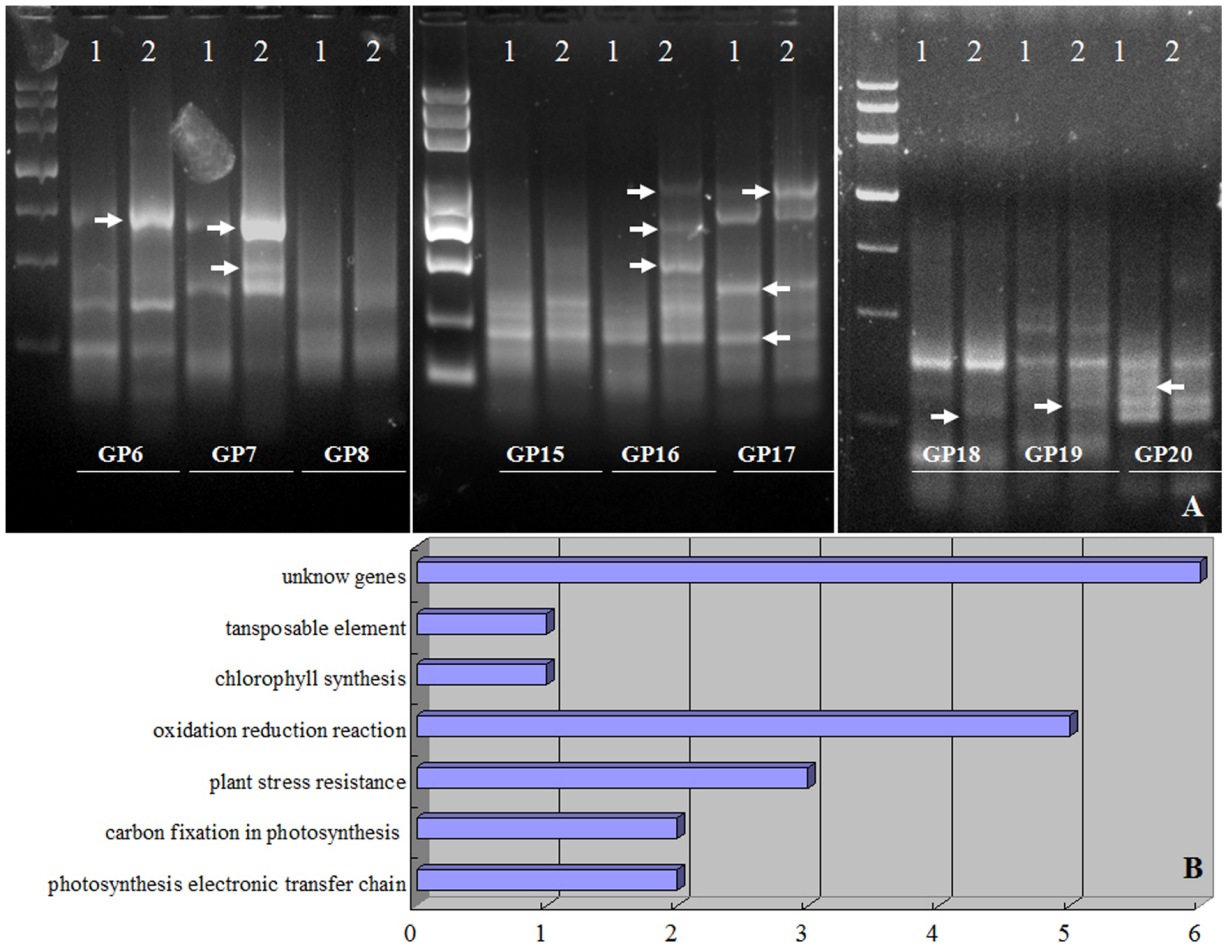
The Genefishing results of leaves and classification of DEGs. A represent the genefishing analysis of leaves that cultivated in sandstone soil (1) and calcareous soil (2), DEG6, DEG7, DEG8, DEG15, DEG16, DEG17, DEG18, DEG19 and DEG20 was the name of different primers; B represent the classification of all the DEGs.

GeneFishing PCR was performed on *L. confusa* that cultivated in calcareous and sandstone soils by using dT-ACP2 and 20 pairs of random primers, respectively. The PCR products were run on 2% agrose gel and these differentially expressed PCR products were cloned into a TOPO TA cloning vector and followed by sequence analysis (Fig. 3A). Totally, 23 DEGs were observed and which could be classified into 7 groups according to their functions, including DEGs involved in photosynthesis electron transfer chain, carbon fixation in photosynthesis, oxidation reduction reaction, plant stress resistance, chlorophyll synthesis, transposable element and some unknown genes (Fig. 3B, Table 1). Most of these DEGs were highly expressed in leaves of *L. confusa* that cultivated in calcareous soil. The main DEGs were described as fellows. DEG3 (RuBisCO activase, Rca) and DEG5 (glycerlde-3-phosphate dehydrogenase, GAPDH) are the genes involved in carbon fixation in photosynthesis [22]. DEG21 (Photosystem I reaction center subunit IV) and DEG23 (photosynthetic electron transfer c, PETC) are genes that are directly involved in the photosynthesis electron transfer chain. Those four DEGs were all up-regulated in the leaves of *L. confusa* that cultivated in calcareous soil, which could improve Pn and promote carbon fixation in photosynthesis or induce more production of ATP in *L. confusa* cultivated in calcareous soil. DEG17 (SAM synthase), DEG18 (NADH dehydrogenase subunit 2 homology), DEG24 (Hydroxy acid oxidase, HAO) and DEG20 (SIUPTG1) are the DEGs involved in oxidation-reduction reaction and abiotic resistance. DEG17 plays an important role in the production of polyamine and the keep mobility of the membrane. It confirms that SAM syntheses might play a role for the adaptability of *L. confusa* to high calcium stresses. DEG18 located in the upper respiratory chain and transfers electrons to NADH ubiquinone and creates the transmembrane proton gradient to synthesize ATP. The results suggest that photorespiration is proportional to calcium concentration when *L. confusa* is faced with higher calcium content. DEG24 and DEG20 encode HAO and SIUPTG1, which are relate with abiotic resistance were also found.

**Table 1:** Blastn analysis of DEGs that involved in photosynthesis in different cultivated conditions.

DEG	Accession	Description	Max score	E value	Max ident
3	HM773394.1	*Musa* AB Group RuBisCO activase (Rca) mRNA, partial cds	138	2e-29	80%
5	XM_002519612.1	*Ricinus communis* (S)-2-hydroxy-acid oxidase, putative	188	1e-44	82%
17	L36680.1	*Pisum sativum* S-adenosylmethionine synthase mRNA	322	1e-84	86%
18	AY059007.1	*Maticora bivirgata* NADH dehydrogenase subunit 2 gene, complete cds; mitochondrial gene for mitochondrial product	41.0	2.8	83%
20	AY622990.1	*Lycopersicon esculentum* UDP-glucose: protein transglucosylase-like protein SlUPTG1 mRNA, complete cds	66.2	1e-07	88%
21	XM_002521115.1	*Ricinus communis* photosystem I reaction center subunit IV A, chloroplast precursor, mRNA	134	2e-28	88%
23	NM_178964.2	*Arabidopsis thaliana* PETC (Photosynthetic Electron Transfer C)	266	2e-67	79%
24	XM_002534085.1	*Ricinus communis* Thioredoxin H-type, mRNA	141	1e-30	75%

**Figure 4:**
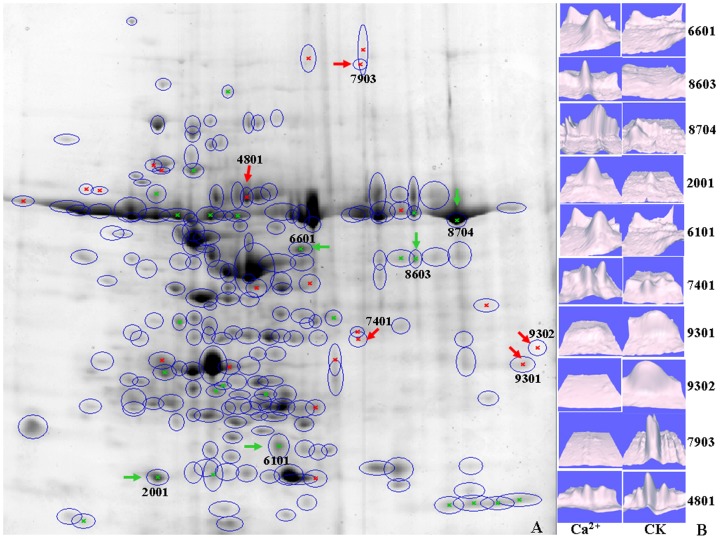
2-DE analysis of *L. confusa* under different cultivated conditions. A represent the 2-DE map that showed the up and down expressed proteins in *L. confusa* that cultivated in calcareous soil (green and red arrow represent the protein that up-regulated and down-regulated in calcareous soil, respectively); B represent the three-dimensional profiles of the individual spots comparing control and Ca^2+^ treated profiles of each of the ten protein spots that showed significant changes.

2-DE was used to identify the DEPs in *L. confusa* that planted in calcareous soil and sandstone soils, respectively (Fig. 4). In all, 25 DEPs expressed more than 2 fold higher and 22 DEPs expressed less than 2 fold lower in calcareous soil were observed (Fig. 4A). The most significant 15 DEPs were performed for MALDI-TOF-MS-MS analysis and 10 DEPs were successfully identified (Table 2). Some up-regulated DEPs and up expressed gene were matched well base on the results that obtained by GeneFishing analysis. For example, the DEPs that identified as RuBisCO large subunit and RuBisCO large polypodiodes formosana subunit were increased about by 14 times (6601) and 27 times (8704), respectively (Fig. 4B). The expression of some DEPs that significantly decreased in calcareous soil environment was also observed (Fig. 4B). Among them, mRNA binding protein precursor decreased by 50%, cell cycle regulated protein 2 (CDC2) homolog decreased by 40%, Zinc finger domain-containing protein (ZBED) decreased by 90% and malate dehydrogenase (MDH) and protochlorophyllide reductase A (PORA) decreased to almost invisible. The highly expressed MDH indicates an increase the E of *L. confusa* that cultivated in sandstone soil. The cell division might be more active in the *L. confusa* that cultivated in sandstone soil for CDC2 were highly expressed.

**Table 2:** The identity of DEPs of *L. confusa* that cultivated in different conditions.

Number	Accession number	The type of proteins	Molecular weight	PI	The relative contents of protein	
					sandstone soil	calcareous soil
6601	gi|8117180	RuBisCO large subunit	50625.5	6.37	234.4	3287.2
8603	gi|1707878	aminomethyltransferase	44248.7	8.77	0	1856.7
8704	gi|131971	RuBisCO large polypodiodes formosana submit	50910.8	6.23	332.4	9089.2
2001	gi|38344034	peroxiredoxin	28604.6	5.17	1902.9	5726.7
6101	gi|4490714	kinesin-related protein katB	84249.8	5.48	399.2	4515.4
7401	gi|26453355	mRNA binding protein precursor	43913.3	7.1	2331.3	1295
9301	gi|1170897	Malate dehydrogenase, glyoxysomal precursor	37714.9	8.82	4076.8	1686.3
9302	gi|15239574	Protochlorophyllide reductase A	43835.6	9.42	5517.6	0
7903	gi|42566188	Zinc finger domain-containing protein	73118.9	5.39	2769.2	25.3
4801	gi|1168812	cell cycle regulated protein 2 homolog (p34cdc2)	10664.7	8.82	8121.5	3033.3

**Figure 5:**
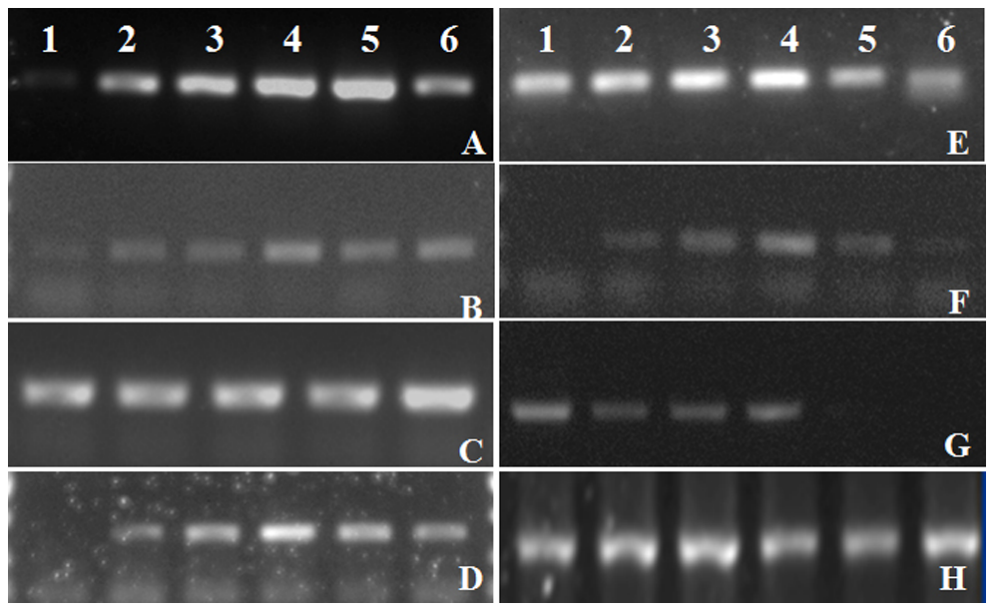
The RT-PCR analysis of DEGs in the leaves of *L. confusa* that treat with different concentration of Ca^2+^. A to H represent the RT-PCR analysis of DEG1, DEG3, DEG5, DEG17, DEG21, DEG23, DEG24 and 18s rRNA, respectively. (1 to 6 represent the *L. confusa* was treated with 0 mg/L, 25 mg/L, 50 mg/L, 75 mg/L, 100 mg/L, 125 mg/L calcium chloride, respectively).

Some DEGs or DEPs were selected out for RT-PCR analysis in the *L. confusa* that treated with different content of calcium (Fig. 5). The results revealed that the expression of DEG3 (Rca) and DEG5 (GAPDH) was improved with increasing concentration of Ca^2+^, which is consistent with the Pn value of *L. confusa* that cultivated in calcareous soils. The expression of most DEGs were first increased with the increasing Ca^2+^ treatment no more than 75 mg/L, and then began to decrease when Ca^2+^ were more than 75 mg/L (for example, DEG17, DEG18, DEG21 and DEG23), this indicated that the expressions of most genes were inhibited in *L. confusa* when the Ca^2+^ content were higher than the calcareous soil. Few DEGs expression were decreased with the increased Ca^2+^ treatment, such as DEG24 that coded as thioredoxin H-type, which functions as a disulfide oxidoreductase and involved in lots of redox dependent cellular processes.

### The interaction between DEPs or DEGs with calcium sensor proteins and other genes

**Figure 6:**
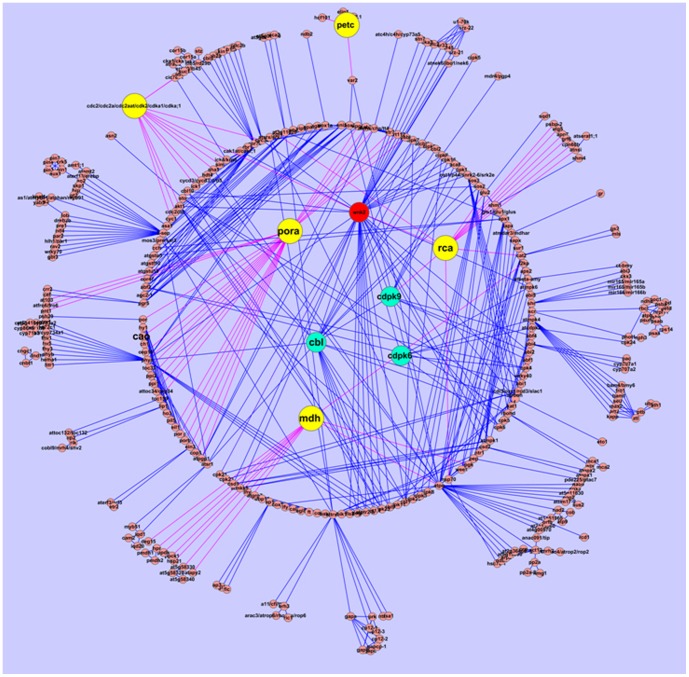
The network of DEGs or DEPs with calcium sensor proteins by using Cytoscape software. Yellow and green cycles represent the DEGs or DEPs and calcium sensor proteins, respectively; the pink lines represent the directly interacted genes with DEGs or DEPs.

A better understanding of the calcium signaling network and the way for the genes that calcium regulated would be very important. Some genes have calcium binding site and could be regulated by calcium directly, and some genes were affected through calcium sensor proteins, such as calcium-dependent protein kinases (CDPKs), calmodulin (CaM) and calcineurin B-like (CBL) proteins [23], [24]. Analyzing the interaction between DEGs with calcium sensor proteins would provide valuable information. Complicated network with 574 nodes and 983 edges was constructed using the Cytoscape software, and some of the DEGs or DEPs (for example, Rca, MDH, CDC_2_, PORA and PETC) that directly or indirectly connected with calcium sensor proteins were observed (Fig. 6). Form the network, we deduced that the DEG3 (RuBisCO activase, Rca) was directly activated by the calcium sensor protein CDPK, in the meanwhile, the activation of CDPK were dependent on both Ca^2+^ and ATP. These results indicated that the activity of CDPK was improved by relatively higher calcium and then enhanced the expression of RuBisCO activase in the leaves, which would improve the carbon fixation and the Pn in *L. confusa* in calcareous soil. WNK2 was a cytoplasmic serine-threonine kinase, which would be active when its calcium-binding C2 domains was bound with Ca^2+^, the CDC2 and PORA were both regulated by WNK2. Other calcium sensor proteins that indirectly interacted with DEGs and DEPs were shown in Fig. 6. The network results indicated that some of DEGs and DEPs were surely affected by calcium or calcium sensor proteins.

## Discussion

The calcium content in calcareous soil was three to four times than that in non-karst soil [5], [14]. A suitable concentration of calcium is required to maintain cell wall structure and membrane function and for photosynthesis [17], [25]. Ca^2+^ depletion in soil may influence carbohydrate storage, photosynthesis, chlorophyll content and antioxidant enzyme activity [15], [16]. The relative Chl content is one of most important factor in determining the Pn [26]. It demonstrated that low concentration of Ca^2+^ could slightly promote Chl accumulation [27], other research revealed that supplementary Ca^2+^ could ameliorate the negative effects of salinity on chlorophyll and dry mass production in strawberry [28]. The present results showed that the relative Chl content and Pn in *L. confusa* planted in calcareous soil were both higher than those planted in sandstone soil. The Chl was synthesized in chloroplast, when plant were exposed to light that higher than those required for photosynthesis, reactive oxygen species are generated in the chloroplasts and cause photodamage [29]. Plants have developed several protective mechanisms when facing the photodamage, one is chloroplast avoidance movement which actually has a role in reducing light absorption by photosystems under high light [29]. Exogenous Ca^2+^ inhibited the loss of chlorophyll under heat stress possibly by its reducing photo-oxidation or maintaining membrane integrity [30], Ca^2+^ treatment also could increase the synthesis of HS proteins, such as HSP26 and HSP70, which could protect the cells and tissues from damage after heat stress [31]. Our present research revealed the relative Chl content of *L. confusa* planted in calcareous soil changed very little compared with those planted in sandstone soil, indicating that the calcium has some effect on eliminating the reactive oxygen species and maintaining chloroplast stability [32], which was in accord with the higher expressed DEP of HAO (hydroxy-acid oxidase) in the *L. confusa* that planted in the calcareous soil.

Ca^2+^ plays a critical role in responding to environmental signals, activating or inactivating the expression of photosynthesis related genes [33], it was also suggested that Pn could be improved by CaCl_2_ in heat stressed plants [19]. CaCl_2_ pretreatment could improve RuBisCO activity under lower temperature, and its higher activity was associated with higher Pn [19], [25], Previous study also revealed that Pn was inhibited by moderate heat stress due to the decrease of the activation of RuBisCO [34]. The present results showed that the Pn of *L. confusa* planted in calcareous soil was higher than that planted in sandstone soil at relatively higher temperature. Some up-regulated DEGs in *L. confusa* planted in calcareous soil were identified as RuBisCO activase (Rca) and glycerlde-3-phosphate dehydrogenase, which were considered to be the key limiting factors in recycling of mitochondrial CO_2_ for carbon fixation in chloroplasts, this results indicated that relatively higher Ca^2+^ could improve photosynthesis and increase carbon fixation by improve the activity of Rca and GAPDH. Some DEPs in *L. confusa* planted in calcareous soil were also identified as RuBisCO large subunit and RuBisCO large polypodiodes formosana subunit. Ca^2+^ addition could efficiently protect chrysanthemum leaves from the damage in photosynthetic apparatus under short term high temperature stress, and the Pn and PS II electron transport were increased by 31.11% and 21.88%, respectively [35]. Tan et al (2011) revealed that CaCl_2_ pretreatment greatly increased PI_ABS_ under heat stress, they deduced that CaCl_2_ pretreatment might play an important role in repairing the PS II complex and maintaining higher photosynthetic activity [19]. Some potential chloroplast targets of CaM could regulate include PSAN (a subunit of PSI) as well as the chloroplast homologue of CPN10 (Chaperonin 10), which was involved in the assembly of ribulose-1,5- bisphosphate carboxylase/oxygenase, thereby providing a further association of calcium regulation to CO_2_ fixation [36], [37]. Two up-regulated DEGs involved in photosynthesis electron transfer chain identified as PsaE and PETC were observed in *L. confusa* planted in calcareous soil, which further confirmed that higher Ca^2+^ in calcareous soil could be conducive to photosynthesis in higher temperature. Nunes-Nesi *et al* (2005) revealed that suppression of mitochondrial malate dehydrogenase in tomato plants leads to an unexpected increase in the rate of photosynthesis, which was consistent with low expression of DEPs that coded as malate dehydrogenase in *L. confusa* planted in calcareous soil [38].

Stomata form pores on leaf surfaces that regulate the uptake of CO_2_ for photosynthesis and the loss of water vapor during transpiration [39]. Free cytosolic Ca^2+^ were increased in response to a high extracellular Ca^2+^ level through a CAS signalling pathway and finally leads to stomatal closure [40]. Present research revealed that E and Gs were decreased in *L. confusa* planted in calcareous soil, which indicated that long-term Ca^2+^ transients and lower malate more likely enhance stoma closure in the leaves of the *L. confusa*, which was also verified by SEM analysis on stomata status and highly expressed HAO and MDH in the *L. confusa* that cultivated in calcareous soil and sandstone soil, respectively. Cell-type specific changes in cytosolic calcium levels were observed in *Arabidopsis* root cells in response to drought [41], *SNAC1* play a important role in environmental stresses [32], the strong induction of *SNAC*1 in plants guard cells suggested that the increasing stomata closure is possible regulated by *SNAC*1, which could also reduce the water loss with increasing stomatal closure [42]. Increasing the expression of SAM synthase could improve the resistance to the chilling and salt stress [43], the plant photosynthesis is restrained when facing salt stresses, the expression of enzymes related with photorespiration is up-regulated to meet the energy requirements [44]. In this study, the highly expressed DEGs that coded as *SNAC* and SAM synthase were significantly induced when planted in calcareous soil, which indicated the higher abiotic resistance of *L. confusa* that planted in calcareous soil. The CI in leaves of *L. confusa* planted in calcareous soil was lower than that of planted in sandstone soil, it indicated more CO_2_ was fixed, which was correlated with higher activity of RuBisCO in leaves of *L. confusa* planted in calcareous soil [19]. The stomata and non-stomata limitation during the diurnal course of photosynthesis was determined by the development trends of intercellular CO_2_ concentration and Gs [45], [46].

Calcium sensor proteins (such as, CDPK, CBL and CAM) were discovered to play a crucial role in abiotic stress signaling in plants, stimulus-specific Ca^2+^ signatures are decoded by Ca^2+^ binding proteins that function as Ca^2+^ sensors [47]–[49]. Qiu *et al* (2007) revealed that a higher level of CDPK activity occurred concurrently with the accumulation of photosynthetic enzymes [50], further study also showed that some transcripts linked to photosynthesis were observed when modulate the CDPK activity in sorghum [51]. The present network revealed that Rca was directly regulated by the downstream of Ca^2+^ target protein CDPK, it indicated that when Ca^2+^ was bound to Ca^2+^ sensors, the CDPKs would change their conformation and interact with RCA to regulate the Pn. ATP and NADPH synthesis via linear photosynthetic electron transfer or solely ATP production via cyclic electron flow was important in photosynthesis [52], the chloroplasts contribute to cellular Ca^2+^ signaling via the chloroplast-localized Ca^2+^ sensor protein CAS [48]. Petroutsos *et al* (2011) also demonstrated that CAS and Ca^2+^ are critically involved in the regulation of the high light response and particularly in the control of LHCSR3 expression [53]. The DEGs that confirmed as PETC and ND2 were observed in the present study, it indicated the high level of Ca^2+^ could bind to Ca^2+^ sensor protein and further improve the activity of PETC and ND2 in the leaves of *L. confusa*. The DEPs of ZBED and PORA were both highly expressed in *L. confusa* cultivated in the sandstone soil, and their functions in *L. confusa* cultivated in sandstone soil remained to be investigated in the future studies. The DEGs or DEPs that observed in this study had close relationship with photosynthesis regulation and calcium signaling could enrich the knowledge of plant adaptation to karst environments.

## Materials and Methods

### Plant materials and growth conditions

The *L. confusa* cultivars were taken from Nongla Karst Experimental Site, Institute of Karst Geology, Chinese Academy of Geological Science (108°19′ E,23°29′ N). The plants were cultivated in the greenhouse and were divided into two groups (15 plants per group): one group was transplanted into Ca^2+^-rich calcareous soil that directly transported from Nongla Karst Experimental Site (Ca^2+^ content 3.1±0.05 g per 100 g soil, the pH value is 7.8±0.2), another group was transplanted into Ca^2+^-poor sandstone soil (Ca^2+^ content 0.02±0.005 g per 100 g soil, the pH value is 6.7±0.3). Average cultivar height is almost 20 cm and one plant was cultivated per pot (The pot diameter is 25 cm). Thus leaves were becoming mature from the seventh leaf onward. During the experimental period from 10 March to 20 July, the average diurnal air temperatures between 19.84°C (day) and 9.5°C (night), respectively, with air humidity fluctuating between 45% and 85% average [8]. The mature leaves were used for photosynthesis, relative chlorophyll content in vivo. The same development stage mature leaves from each group were cut and mixed together, and transferred to liquid nitrogen immediately for Genefishing PCR and proteomics analysis.

### Photosynthesis and relative chlorophyll measurement

Fully expanded mature leaves cultivated in Ca^2+^-rich calcareous soil and Ca^2+^-poor sandstone soil were sampled for measurement, respectively. The measurements were taken from 08:00 AM to 18:00 PM in July on plants under clear sky ambient sunlight, the values stated are mean values from three days. The fully expanded leaves of five different plants by using an open system photosynthetic gas analyzer (PP Systems Inc. model TPS-1, Amesbury, MA, USA) to measure the net photosynthesis rate (Pn), stomata conductance (Gs), transpiration rate (E) and intercellular CO_2_ concentrations (CI) of plants in the greenhouse [54]. The chlorophyll content was measured (10 repeats per leaf) by using the CL-01 chlorophyll content meter, which determines relative chlorophyll content using dual wavelength optical absorbance (620 and 940 nm) measurements from leaf samples (Hansatech Instruments, Norfolk, UK) [55], [56]. The measurement Data were analysed using SPSS version 12.0 statistical software. Probability (p) values of <0.05 were considered significant. The graph data were processed using origin 7.0 software (Microcal Software, Inc.,Northampton, MA, USA).

### Transmission electron microscope (TEM) and scanning electron microscope (SEM) analysis of leaves

For TEM analysis, mmature leaf samples of *L. confusa* that cultivated with Ca^2+^-rich calcareous soil and Ca^2+^-poor sandstone soil were collected and washed with phosphate buffer solution, cut into 0.5 cm×0.5 cm slices and immediately immersed for fixation in 2.5% glutaraldehyde (v/v, pH = 7.8) with 0.2 M cacodylate and 2% (w/v) buffered osmium tetroxide. The samples were dehydrated through a graded ethanol series (30%, 50%, 70%, 85%, 95%, 100%) for 10 min each and in 100% isoamyl acetate twice for 20 min. Tissues were then vacuumed, incubated for 4 hours at 4°C and fixed with 2% potassium pyroantimonate (pH = 7.8) for 16 hours at 44°C. Tissues were then rinsed with PBS solution, dehydrated with graded acetone solution, embedded with Spurrs epoxy resin and sectioned with superfine section machine. For the calcium positioning, the slices were incubated in 100 mM EGTA (pH 8.0) solution at 60°C for 1 hour and stained with uranium acetate for observation followed by Tian *et al* [57].

For SEM analysis, mature leaves of *L. confusa* samples were first treated through 10%, 20%, 30%, 50% and 70% of ethanol and then immersed in 100% acetone twice. The dehydrated samples were treated with CO_2_ critical point dryer. The dried samples were mounted on brass disks coated with Pt under vacuum. The ultrastructure of samples were observed by SEM [8].

### Identification of differentially expressed genes (DEGs) by using GeneFishing PCR

Mature *L. confusa* leaves cultivated in Ca^2+^-rich calcareous soil and Ca^2+^-poor sandstone soil were chosen for RNA extraction using TRIzol (Invitrogen) according to the manufacturer's instructions. The RNA pellets were frozen and stored at −80°C until use. Differential display PCR was performed using the GeneFishing kit according to the manufacturer's instructions (Seegene, Inc.) and which was performed for three replications. The PCR products were separated in 1.2% agarose gel, only the differentially expressed bands that repeated existed in three replications were selected for further sequence analysis. The differentially expressed bands were extracted from the gel using a QIAquick Gel extraction kit (Qiagen) and directly cloned into a pGEM-T Easy vector for sequencing. Semi-quantitative RT-PCR was used for confirmation of the above Genefishing results. 18S rRNA was used as an internal control. The sequence of primers was showed in Table S1.

### Identification of differentially expressed proteins (DEPs) by using two-dimensional gel electrophoresis (2-DE)

Mature leaves of *L. confusa* were collected and immediately freezed and stored at −80°C. For total protein extraction, samples were ground in liquid nitrogen to fine power, and the protein extraction method as outlined by Damerval *et al* [58]. Protein concentration was determined using the RC DC Protein Assay Kit I (Bio-Rad (500-0122), Hercules, CA, USA) with bovine serum albumin as standard according to the manufacturer's instructions. For 2-DE analysis, a volume of 300 µL rehydration buffer containing 1 mg of protein was loaded onto linear pH gradient (IPG) strips (ReadyStrip 170 mm, pH 3–10, Bio-Rad, Hercules, CA, USA). Isoelectric focusing (IEF) was performed by using a Protean IEF Cell (Bio-Rad, Hercules, CA, USA) system and followed their operation manual. The isoelectric focusing system were: 50 V for 1 h, 200 V for 1 h, 500 V for 1 h, 1000 V for 1 h, a linear increase of voltage to 10,000 V for 4 h, 10000 V for 8 h achieving approximately 80 000 Vhr. After IEF, the strips were then equilibrated twice for 15 min each in an equilibration buffer as described [59]. The second dimension separation of proteins was performed on 10% SDS-PAGE gels using a Bio-Rad PROTEIN II xi cell system. After electrophoresis, the gels were stained with colloidal Coomassie brilliant blue (CBB) G-250 according to Candiano et al [60]. The stained gels were scanned with a UMAX Powerlook 2100XL Imaging System with a resolution of 300 dpi and processed using the PDQuest 8.01 software (Bio-Rad Laboratories, Hercules, CA). The spots that changed in abundance more than two-fold and the least significant difference performed more than 95% (*p*<0.05) were selected for protein identification.

The MS/MS analysis (MALDI-TOF) for DEPs were performed as described by Li *et al* [61]. The DEPs were dug from the gels, placed in the 1.5 mL EP tube, washed twice with pure water, and decolorized with the same volume of decoloring liquid (30 mM, 100 mM potassium ferrocyanide and sodium thiosulfate, 100 mM ammonium bicarbonate, pH 8.0) for 20 min; Proteins were then washed with pure water again and dehydrated with acetonitrile, vacuum dried, and was added digested with 20 µL trypsin (20 ng/μL) at 37°C overnight; For dissolution, 50 µL serine extract (50% acetonitrile, 5% trifluoro ethyl) was added for 15 min, repeat twice, vacuum dry and 10 µL 0.1% trifluoro ethyl was added for dissolution. Then mix 2 µl of the above extraction liquid with 2 µl Mass spectrum loading buffer (Alpha-cyanogen-4-hydroxyl, 50% acetonitrile, 0.1% three fluorine acetic acid) and analyzed with MALDI-TOF-MS Autoflex II. The settings were as follows: 20 kV accelerating voltage, 60–65% grid voltage, 160 ns delay, 200/spectrum of the laser make protein point into peptides about 1000–4000 Da and obtain mass spectrometry results. The protein was identified according to peptide fingerprint spectra combined with the MASCOT search engine (http://www.matrixscience.com). Search parameters were set as: taxonomy, Virdiplantae; enzyme, trypsin; max missed cleavages, 1; fixed modification, carbamidometyl (C); variable modifications, oxidation (M); fragment mass tolerance, ±0.2 Da; and mass accuracy, 50 ppm.

### RT-PCR analysis of DEGs and DEPs of *L. confusa* with different concentration of CaCl_2_ treatment

2Hydrochemistry analysis showed that the average concentration of Ca^2+^ in Landiantang spring of karst area of Nongla was 75.20 mg/L in the year of 2003 and 2004 [62]. In this experiment, the successive concentration of CaCl_2_ solution was designed to treat the *L. confusa* plants, and use 75 mg/L as tipping point. Twenty four pots of materials were separated into six groups, washed with pure water for a few times. For RT-PCR analysis of the DEGs that obtained by Genefishing in different CaCl_2_ solutions (treated with 0 mg/L, 25 mg/L, 50 mg/L, 75 mg/L, 100 mg/L, 125 mg/L CaCl_2_ solutions), fresh mature leaves mixed samples for treatment with 36 h were taken and preserved in −80°C fridge immediately. The primers that used for RT-PCR was listed in Table S2. The RT-PCR conditions was as follows, the samples were first treated with 95°C and followed with 30 cycles of 60 s of 94°C denaturation step, 40 s of 68°C annealing step and 40 s of 72°C extension. 18S rRNA was used as an internal control.

### Network analysis of DEGs or DEPs with calcium sensor proteins

All sequences of DEGs were used to perform BLAST analysis. According to the conservatism of the gene, we deemed that DEGs should be the highest homology gene. The functions of DEGs or DEPs were analyzed using Kyoto Encyclopedia of Genes and Genomes (KEGG) database (http://www.genome.jp/tools/blast). The gene interaction between these Ca^2+^ induced DEGs or DEPs with calcium sensor proteins was as follows. The plugin of Agilent Literature Search in Cytoscape software (http://www.cytoscape.org/plugins/index.php) was used.

## Supporting Information

Table S1 **Primers used for GeneFishing** **PCR.**
(DOC)

Table S2 **Primers used for RT-PCR analysis of some DEGs or DEPs.**
(DOC)

Table S3 **The correlation between E, Gs, Pn and environment factors which cultivated in sandstone soil and calcareous soil.**
(DOC)
