# Supplementary material for: Characterization and Molecular Interpretation of the Photosynthetic Traits of Lonicera confusa in Karst Environment
Source: PLoS One. 2014 Jun 24;9(6):e100703. doi: 10.1371/journal.pone.0100703 (PMC4069104; doi:10.1371/journal.pone.0100703)
Supplement: Table S1 — Primers used for GeneFishing PCR. (DOC) [file pone.0100703.s001.doc]

Table S1 Primers used for GeneFishing PCR

| Primer Name | Primer sequence |
| --- | --- |
| dTACP1 | 5’-CTGTGAATGCTGCGACTACGATIIIII(T)_18_-3’ |
| ACP1 | 5’-GTCTACCAGGCATTCGCTTCATIIIIIGCCATCGACC-3’ |
| ACP2 | 5’-GTCTACCAGGCATTCGCTTCATIIIIIAGGCGATGCC-3’ |
| ACP3 | 5’-GTCTACCAGGCATTCGCTTCATIIIIICCGGAGGATG-3’ |
| ACP4 | 5’-GTCTACCAGGCATTCGCTTCATIIIIIGCTGCTCGCG-3’ |
| ACP5 | 5’-GTCTACCAGGCATTCGCTTCATIIIIIAGTGCGCGCG-3’ |
| ACP6 | 5’-GTCTACCAGGCATTCGCTTCATIIIIIGGCCACATCG-3’ |
| ACP7 | 5’-GTCTACCAGGCATTCGCTTCATIIIIICTGCGGATCG-3’ |
| ACP8 | 5’-GTCTACCAGGCATTCGCTTCATIIIIIGATGCCGCTG-3’ |
| ACP9 | 5’-GTCTACCAGGCATTCGCTTCATIIIIIGATGCCGCTG-3’ |
| ACP10 | 5’-GTCTACCAGGCATTCGCTTCATIIIIITGGTCGTGCC-3’ |
| ACP11 | 5’-GTCTACCAGGCATTCGCTTCATIIIIICTGCAGGACC-3’ |
| ACP12 | 5’-GTCTACCAGGCATTCGCTTCATIIIIIACCGTGGACG-3’ |
| ACP13 | 5’-GTCTACCAGGCATTCGCTTCATIIIIIGCAAGTCGGC-3’ |
| ACP14 | 5’-GTCTACCAGGCATTCGCTTCATIIIIIGCAAGTCGGC-3’ |
| ACP15 | 5’-GTCTACCAGGCATTCGCTTCATIIIIICCACCGTGTG-3’ |
| ACP16 | 5’-GTCTACCAGGCATTCGCTTCATIIIIIGTGGACGGTG-3’ |
| ACP17 | 5’-GTCTACCAGGCATTCGCTTCATIIIIICAAGCCCACG-3’ |
| ACP18 | 5’-GTCTACCAGGCATTCGCTTCATIIIIICGGAGCATCC-3’ |
| ACP19 | 5’-GTCTACCAGGCATTCGCTTCATIIIIICTCTGCGAGC-3’ |
| ACP20 | 5’-GTCTACCAGGCATTCGCTTCATIIIIIGACGTTGGCG-3’ |
| dTACP2 | 5’-CTGTGAATGCTGCGACTACGATIIIII(T)_15_-3’ |
