# Supplementary material for: Characterization and Molecular Interpretation of the Photosynthetic Traits of Lonicera confusa in Karst Environment
Source: PLoS One. 2014 Jun 24;9(6):e100703. doi: 10.1371/journal.pone.0100703 (PMC4069104; doi:10.1371/journal.pone.0100703)
Supplement: Table S2 — Primers used for RT-PCR analysis of some DEGs or DEPs. (DOC) [file pone.0100703.s002.doc]

Table S2 Primers used for RT-PCR analysis of some DEGs or DEPs

| DEG No. | Forward primer sequence (5’-3’) | Reverse primer sequence (5’-3’) |
| --- | --- | --- |
| 1 | GGCGAAATCGGAATCATC | AGATGAGCTCGTACACAG |
| 3 | TGCCAACAAGGATGCTAT | ACGATGAAGCGTCAAACA |
| 5 | CACATCGGGAAATCAAAG | GGTCACAACATGCAACAG |
| 17 | CTTATGCCATTGGTGTTC | ATTGTCCGCTAGGAGTTA |
| 21 | GGTGTTTTCATTGGCGGC | CGTCCCAATCAGTGGCAAT |
| 23 | CCTTCGCTGCCTCACTAT | TACACTCCTTTGCCTCCC |
| 24 | CGCTCCATTCCTCGCAGAGTT | AGAACTTGTGAACCTTTA |
| 18S rRNA | GTAACAAGGTTTCCGTAGGTG | ACCACCACTCGTCGTGACGT |
