# Supplementary material for: Characterization and Molecular Interpretation of the Photosynthetic Traits of Lonicera confusa in Karst Environment
Source: PLoS One. 2014 Jun 24;9(6):e100703. doi: 10.1371/journal.pone.0100703 (PMC4069104; doi:10.1371/journal.pone.0100703)
Supplement: Table S3 — The correlation between E, Gs, Pn and environment factors which cultivated in sandstone soil and calcareous soil. (DOC) [file pone.0100703.s003.doc]

Table.S3 The correlation between E, Gs, Pn and environment factors sandstone soil and calcareous soil

| Index | | Environment factors and [physiological factor](http://dict.cnki.net/dict_result.aspx?r=1&t=physiological+factor&searchword=生理因子) | | | | | |
| --- | --- | --- | --- | --- | --- | --- | --- |
|  |  | PAR | Air  humidity | Air  temperature | CO_2_ concentration  of sample room | Leaf  temperature | CI |
| sandstone soil plant | E | 0.565 | -0.411 | 0.441 | -0.596 | 0.358 | -0.315 |
|  | Gs | 0.219 | -0.128 | -0.461 | 0.233 | -0.537 | -0.401 |
|  | Pn | 0.795* | 0.475 | -0.042 | 0.141 | -0.005 | -0.997** |
| calcareous soil plant | E | 0.681* | -0.281 | 0.887** | -0.745* | 0.896** | -0.021 |
|  | Gs | 0.704* | -0.084 | 0.708* | -0.614 | 0.723* | -0.204 |
|  | Pn | 0.655 | 0.546 | -0.210 | 0.351 | -0.123 | -0.969** |

* Correlation is significant at the 0.05 level (2-tailed).

** Correlation is significant at the 0.01 level (2-tailed).
